# Building a public health infrastructure to support family caregivers of people with dementia

**DOI:** 10.1093/geront/gnaf223

**Published:** 2025-10-01

**Authors:** Joseph E Gaugler, Elma Johnson, Gary Epstein-Lubow, Lauren Parker, Fayron Epps, Ashley Millenbah

**Affiliations:** School of Public Health, University of Minnesota-Twin Cities, Minneapolis, Minnesota, United States; Building Our Largest Dementia Infrastructure (BOLD) Public Health Center of Excellence on Dementia Caregiving, School of Public Health, University of Minnesota-Twin Cities, Minneapolis, Minnesota, United States; School of Public Health, University of Minnesota-Twin Cities, Minneapolis, Minnesota, United States; Building Our Largest Dementia Infrastructure (BOLD) Public Health Center of Excellence on Dementia Caregiving, School of Public Health, University of Minnesota-Twin Cities, Minneapolis, Minnesota, United States; Building Our Largest Dementia Infrastructure (BOLD) Public Health Center of Excellence on Dementia Caregiving, School of Public Health, University of Minnesota-Twin Cities, Minneapolis, Minnesota, United States; Education Development Center, Alpert Medical School and School of Public Health, Brown University, Providence, Rhode Island, United States; Building Our Largest Dementia Infrastructure (BOLD) Public Health Center of Excellence on Dementia Caregiving, School of Public Health, University of Minnesota-Twin Cities, Minneapolis, Minnesota, United States; Johns Hopkins Bloomberg School of Public Health, Baltimore, Maryland, United States; Building Our Largest Dementia Infrastructure (BOLD) Public Health Center of Excellence on Dementia Caregiving, School of Public Health, University of Minnesota-Twin Cities, Minneapolis, Minnesota, United States; School of Nursing, University of Texas Health Science Center at San Antonio, San Antonio, Texas, United States; School of Public Health, University of Minnesota-Twin Cities, Minneapolis, Minnesota, United States; Building Our Largest Dementia Infrastructure (BOLD) Public Health Center of Excellence on Dementia Caregiving, School of Public Health, University of Minnesota-Twin Cities, Minneapolis, Minnesota, United States

**Keywords:** Caregiving—informal, Alzheimer’s disease, Evidence-based practice, Home and community-based care and services

## Abstract

The Centers for Disease Control and Prevention-funded Building Our Largest Dementia (BOLD) Infrastructure’s Public Health Center of Excellence on Dementia Caregiving (PHCOE-DC) is one of three national centers designed to help public health departments strengthen and grow their Alzheimer’s disease and related dementia initiatives. The PHCOE-DC specializes in disseminating tools and resources to help public health agencies develop programming and partnerships that support family caregivers of individuals with dementia. Through its reach and dissemination efforts, the PHCOE-DC has helped to elevate dementia caregiving as a priority for public health departments. Since 2020, the PHCOE-DC has increased visibility for the role of public health in strengthening the support infrastructure for family caregivers of individuals with dementia and has established a network of national leaders in dementia caregiving. This article summarizes PHCOE-DC’s past work and potential future activities as the Center continues to elevate dementia caregiving as a priority for public health.

For nearly 50 years, a robust literature has emerged documenting the potentially adverse health effects of providing unpaid care to someone living with Alzheimer’s disease or a related dementia (ADRD) (Alzheimer’s Association, 2024; Brodaty & Donkin, 2009; Gilhooly et al., 2016; Schulz & Martire, 2004). This body of research has helped to inform and elevate dementia caregiving as a matter of both societal and *public health* concern. There are several reasons why dementia caregiving is a relevant issue in public health. Dementia caregiving impacts the health of both caregivers and care recipients within the population. Some members of this population may bear the effects of dementia caregiving unequally. Effective strategies can be employed to reduce these negative impacts (Bouldin et al., 2021; Gaugler et al., 2023). A range of public health actions is possible to enhance the support and well-being of dementia caregiving families, including improved surveillance and identification of ADRD caregivers; building and enhancing community partnerships; advancing dementia-capable health care and related payment incentives; and reducing the stigma of dementia and ADRD caregiving can strengthen support and services for families (Gaugler et al., 2023).

The conceptual model in Figure 1 (from Gaugler et al., 2023) illustrates how public health actions within a social determinants of health (SDOH) framework can impact potential mechanisms that influence dementia caregiver health and well-being. This constellation of public health actions can address various mechanisms and processes that drive caregiving health, such as reducing financial strain, improving health literacy, addressing unmet needs for dementia care, promoting meaningful activity engagement, and reducing social isolation. Although much research is required to test and establish the SDOH and dementia caregiving conceptual model presented in Figure 1, it also offers a potential roadmap for demonstrating how public health interventions can enhance the well-being of dementia caregivers (and likely their care recipients).

**Figure 1.**
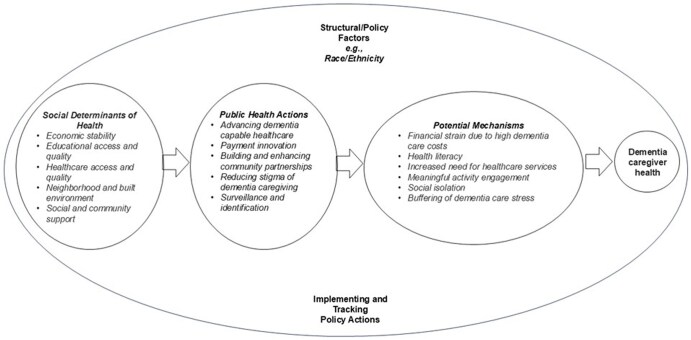
Social determinants of health and dementia caregiving: public health actions and mechanisms (included with permission from Gaugler et al. [2023]).

This conceptual framing of the public health imperative of dementia caregiving (Gaugler, 2022) underpins the operations and mission of the Building Our Largest Dementia Infrastructure (BOLD) Public Health Center of Excellence on Dementia Caregiving (PHCOE-DC). As the initial 5-year funding cycle of the PHCOE-DC comes to an end, this paper summarizes the goals and mission of the Center; the Center’s activities and initiatives to date to engage with public health agencies and advance their work in supporting dementia caregivers; and a summary of future initiatives that will advance PHCOE-DC’s efforts to establish dementia family caregiving as a public health imperative.

## The goal and mission of BOLD and PHCOE-DC

One challenge to elevating dementia and dementia care as a public health priority is the “siloing” of dementia initiatives (Gaugler, 2022). In many states and communities, aging service providers (e.g., Aarea Agencies on Aging) and, to some extent, healthcare systems are primarily responsible for delivering dementia services, programming, and strategy, with health departments traditionally playing a more limited role. One reason for this structural arrangement is funding, where dementia care services and support resources are channeled to aging service networks and less to departments of health (e.g., Older Americans Act funding). Therefore, the extent of collaboration and partnerships on issues related to dementia and dementia care between departments of health (either at the state or local levels), healthcare systems, and aging service providers varies widely across states.

As the introductory article to this supplemental issue by McGuire (2025) summarizes in detail, the Building Our Largest Dementia Infrastructure (BOLD) for Alzheimer’s Act aims to advance the role of public health in reducing dementia risk, early detection of dementia, and supporting caregivers of individuals with dementia. Passed into law on December 31, 2018 (P.L. 115-406), the BOLD Act has helped to establish a public health infrastructure to support health departments at the state, local, and tribal levels. Moreover, the BOLD Act directed the Centers for Disease Control and Prevention (CDC) to establish three national centers of public health excellence in dementia, one of which focuses on supporting dementia caregivers through public health dissemination and engagement.

The BOLD PHCOE-DC was launched in 2020 to support state, local, territorial, and tribal public health agencies across the United States in developing strong dementia caregiving programming and initiatives. Through collaboration and various dissemination strategies, the PHCOE-DC aims to support state/local BOLD initiatives (as well as non-BOLD agencies and organizations) that ultimately improve the quality of life for people living with dementia and their caregivers. Below, we describe the Center’s structure, the dissemination and engagement activity of the PHCOE-DC since its founding, and the potential next steps for the Center and BOLD in its ongoing efforts to advance public health action in dementia and dementia care.

## PHCOE-DC structure

**Figure 2.**
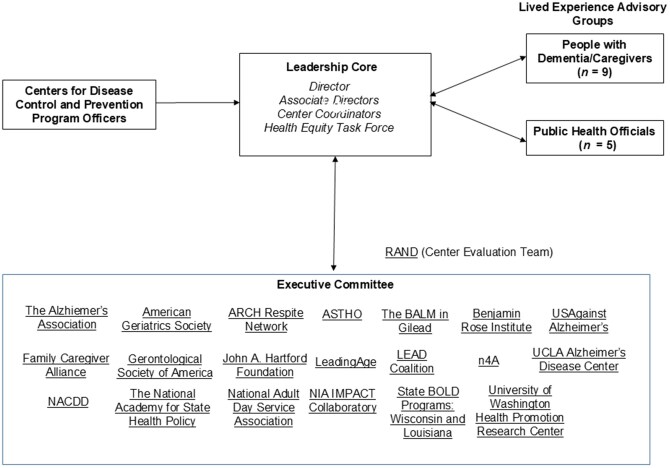
BOLD Public Health Center of Excellence on Dementia Caregiving administrative structure.

The structure of the PHCOE-DC is shown in Figure 2. The Center includes a Leadership Core, consisting of the Director and Associate Directors, and, as described below, a Health Equity Task Force. The Health Equity Task Force (HETF) features members from the Johns Hopkins Bloomberg School of Public Health, the University of Texas Health Science Center at San Antonio, the University of Virginia, and the University of Minnesota. Previous members represent caregiver-focused organizations such as the Diverse Elders Coalition and the National Alliance for Caregiving. Given the central focus of the PHCOE-DC on health equity, members of the HETF also serve on the center’s Leadership Core.

An essential aspect of the PHCOE-DC is its Executive Committee, which includes an extensive network of national leaders representing organizations and initiatives that have championed research, service innovation, and policy advancement in caregiving. Also part of the Center are two Lived Experience Advisory Groups (LEAG), composed of public health officials and dementia caregivers from diverse communities, care contexts/settings, and backgrounds. The LEAGs contribute to, review, and lead efforts to ensure that the perspectives of public health agency providers, people living with dementia, and dementia caregivers are incorporated in Center dissemination activities (see Kline et al., 2025).

## Public health dissemination strategy of the PHCOE-DC

The PHCOE-DC's primary objective is to engage with public health agencies to assist them in identifying and advancing their activities to best support dementia caregivers. The Center achieves this goal through three dissemination approaches: (1) webinars, a national conference, and a community of practice that examine topics and public health strategies to support dementia caregivers as well as build partnerships across states and public health agencies, (2) toolkits designed to support public health agencies in implementing their caregiving strategies, and (3) peer-reviewed manuscripts. Highlights from each of these dissemination activities are summarized below.

Since its founding in 2020, the Center has hosted 30 individual webinars, including four webinar series and numerous virtual panel discussions featuring various subject matter experts, public health practitioners, and leaders in dementia and caregiving. These webinars have reached over 6,700 attendees and addressed various topics to support public health action, including partnership building, workforce development, public health data sources (all Public Health Actions in Figure 1), and the dissemination of caregiving programs. Related to cross-sector partnerships, webinars have included topics such as the implementation of the Recognize, Assist, Include, Support, & Engage (RAISE) National Strategy to Support Family Caregivers (RAISE Act Family Caregiving Advisory Council & The Advisory Council to Support Grandparents Raising Grandchildren, 2022) by departments of public health (see Implementing and Tracking Policy Actions in Figure 1), the identification of family caregivers across care settings, and opportunities for partnership between public health departments and faith-based organizations (FBOs) in dementia-related outreach and education (both Public Health Actions in Figure 1). In the domain of workforce development, multiple virtual events showcased the collaboration between public health departments and Geriatric Workforce Development Programs across the country. Others highlighted opportunities for public health to support the development and training of a dementia-capable community health worker (CHW) workforce. In the domain of public health data, Center webinars have explored the potential of state dementia registries and other innovative approaches, such as crowdsourcing (see below), for creating new sources of caregiver data to inform public health action. To support the dissemination of effective caregiving programs, webinar topics have addressed the cultural adaptation of caregiving programs and advancing health equity in dementia caregiving through public health policy and practice, among others (The BOLD Public Health Center of Excellence on Dementia Caregiving, 2025a) (see Structural/Policy Factors in Figure 1).

The PHCOE-DC has also sought to create a “community of practice” for public health professionals to share successes, lessons learned, and innovative approaches to supporting family dementia caregivers. To that end, the Center established a bi-monthly virtual roundtable series to feature the caregiving work of different state health departments. The 13 roundtables hosted by the Center were attended by 1,623 individuals. They provided a space for dialogue and an interactive forum for health agencies to share and learn from one another’s work, inspiring and driving public health action to support family caregivers nationwide. In addition to the roundtable series, the Center hosted *The Public Health Opportunities and Challenges of Dementia Caregiving Conference* in Minneapolis, Minnesota, in the Spring of 2022. The first national conference of its kind, this event brought together over 500 attendees and numerous national leaders in policy, public health practice, and research on dementia caregiving. The variety of topics presented, along with their applicability and relevance to public health practice, was identified as a major strength by attendees (an earlier version of Figure 1 guided the content and activities of the conference). Over 90% of participants who provided event feedback reported that the information shared was pertinent to their work and aligned with the needs of their organizations. Among respondents from public health agencies, 94% found the information actionable, and 58% anticipated no barriers to implementing the tools introduced during the conference.

Over the past 5 years, the Center has published seven toolkits to provide practical guidance, tools, and resources that public health agencies can utilize in their caregiving efforts. Toolkits are designed to support agencies in implementing different caregiving strategies, including dissemination of evidence-based programs for caregivers, selecting public health strategies on caregiving, building partnerships with FBOs, supporting the growth of memory cafés, and culturally adapting programs to serve dementia caregivers in racially and ethnically diverse communities, among others (again, key Public Health Actions in Figure 1) (The BOLD Public Health Center of Excellence on Dementia Caregiving, 2025b).

The PHCOE-DC has also utilized peer-reviewed publications to augment its public health dissemination efforts. Its publications aim to promote and translate research findings into practical recommendations for public health actions that support family caregivers of individuals with dementia. In its four peer-reviewed publications to date, the Center has identified policy solutions that can establish and sustain caregiving as a public health priority across the United States (Gaugler, 2022; cited 25 times per Google Scholar, July 2025), highlighted public health actions to address social determinants of health affecting the well-being of family dementia caregivers (Gaugler et al., 2023; cited 23 times per Google Scholar, July 2025), showcased a promising model for integrating people with lived experience in the planning of public health strategies related to dementia and caregiving (Kline et al., 2025) and identified opportunities for public health leadership in developing a dementia-capable CHW workforce (Johnson et al., 2025). At the time of this writing, the Center is working on an additional manuscript that highlights the need for more localized caregiver data to better tailor services for caregivers in local communities.

To further build public health capacity, the PHCOE-DC has provided free technical assistance to health agencies for selecting and implementing their caregiving goals. Thanks to its large network of national organizations and leaders in dementia caregiving, the Center has been able to leverage the expertise of its members and provide tailored support and guidance to public health departments. Since 2020, the PHCOE-DC has received 45 requests for technical assistance from health departments, aging service providers, and other partners. The Center has assisted with analyzing the return on investment and cost-effectiveness of evidence-based programs for family dementia caregivers, recommending evaluation metrics for caregiver interventions, selecting caregiver assessment tools, and creating tailored social media messages using state-specific Behavioral Risk Factor Surveillance System (BRFSS) Caregiver Module data, among other topics.

Finally, Center leadership has presented and promoted the PHCOE-DC’s work at 37 different national and state conferences, organizations, and meetings, reaching an additional 3,400 people.

### Health Equity Task Force

Health equity is central to the PHCOE-DC and is interwoven throughout all Center activities. The main objective of the HETF is to ensure that issues related to health disparities in dementia caregiving are integrated and addressed in all dissemination activities of the PHCOE-DC. For example, the HETF ensures that cultural tailoring and adaptation are applied to messaging disseminated by the PHCOE-DC regarding the importance of dementia caregivers. The health equity focus of the Center also ensures that its dissemination process incorporates necessary cultural adaptations. Hence, the Center reaches diverse user organizations (e.g., see the description of public health dissemination of the Alter program below).

The HETF participates in all Center activities to prioritize content, issues, and adaptations central to reaching underrepresented populations such as Black/African American, Hispanic/Latino, Native American/American Indian, LGBTQ, and intellectual and developmental disability dementia caregivers. Since its inception, the HETF has hosted a range of webinars and symposia to disseminate information on caregiver support. The HETF has also developed two toolkits: “A Public Health Agency’s Guide to Partnering with Faith-Based Organizations” and “Cultural Adaptation and Cultural Tailoring of Dementia Care Supports,” to further support dissemination efforts. The HETF launched a mentorship initiative, developed in collaboration with faculty leads from Emory University and Johns Hopkins University, to engage students from Historically Black Colleges and Universities and Hispanic-Serving Institutions. This program aimed to provide underrepresented students with meaningful research experiences, professional development, and mentorship to support their career trajectories and academic growth.

#### Health equity-themed webinars

Webinars have been essential in disseminating knowledge about disparities in dementia care and promoting approaches to address such disparities. The HETF launched its first series, *Cultural Adaptations in Dementia Caregiving* (see The BOLD Public Health Center of Excellence on Dementia Caregiving, 2025a, for all HETF resources). This three-part installment brought together experts to discuss tailoring dementia caregiving interventions for culturally diverse communities, including Black/African American, Hispanic/Latino, Pacific Islander, Native American, and Alaskan Native populations. Each session highlighted the unique caregiving challenges faced by these communities and emphasized the importance of culturally tailored support that addresses specific cultural contexts, enhances caregiving efficacy, and ensures interventions are more accessible and impactful across diverse populations.

Further expanding the conversation on community-based support, the HETF developed *Faith and Public Health*, another three-part webinar installment that explores the role of FBOs in dementia caregiving. Recognizing FBOs’ longstanding involvement in health promotion and disease prevention, this series examined how FBOs serve as crucial public health partners in dementia caregiving. Sessions highlighted the dementia-related work FBOs are engaged in, their roles as public health agents, and strategies for public health agencies to build effective partnerships with these organizations.

Additionally, the HETF hosted a Health Equity Webinar on the application of health equity principles in dementia caregiving, focusing on both policy and practice. This session detailed actionable approaches to applying health equity frameworks in dementia care policy and community-level interventions. Through these initiatives, the HETF reaffirms its commitment to addressing health disparities and enhancing dementia care for historically underserved communities.

#### Alter as a HETF initiative

Recognizing the significant value of having trusting relationships with FBOs and places of worship in minority racial and ethnic communities, the HETF supported the specific efforts of the Alter™ program to address dementia-related disparities in the Black/African American community. The Alter program involves partnering with Black/African American faith communities to establish dementia-friendly congregations and create culturally grounded, supportive, informed, and spiritually enriching spaces for families affected by dementia (Epps et al., 2022). The Alter™ program seeks to create supportive environments for families experiencing dementia by critically examining and transforming traditional practices within Black/African American faith communities. By addressing a range of SDOH and structural inequities, the program advances equitable access to brain health services, dementia care, and community-based support. Grounded in principles of health equity and cultural relevance, the Alter™ program is designed to empower under-resourced communities through the intentional restructuring of existing systems within Black/African American congregations. The program is offered at no cost, providing faith leaders and congregants with comprehensive training, facilitated access to supportive resources, and financial assistance to foster sustainable, dementia-friendly infrastructure within their ministries.

Members of the HETF participated in strategic meetings to explore opportunities for scaling and expanding the Alter program. A primary outcome was the development of “A Public Health Agency’s Guide to Partnering with Faith-Based Organizations” and “Alter™—Public Health Agency Education and Dissemination Toolkit,” two complementary toolkits that, together, support public health partnerships with FBOs and provide the framework for supporting Black/African American dementia-friendly congregations (The BOLD Public Health Center of Excellence on Dementia Caregiving, 2025b). These toolkits are step-by-step guides to support public health agency staff in training, understanding the community, recruiting FBOs, disseminating Alter programming, and partnering with other local community resources. Ultimately, the work between Alter™ and PHCOE-DC empowers public health agencies to support Black/African American older adults and caregivers affected by dementia by fostering resilience and enhancing overall well-being.

#### Lived Experience Advisory Groups

Along with the HETF, the Center’s LEAGs further help the PHCOE-DC incorporate the voices of diverse groups and communities into its work. The Center has two advisory groups: the LEAG, composed of nine current and former family dementia caregivers of diverse ethnic and racial backgrounds, and the Public Health Official LEAG, which includes five representatives from BOLD-funded state programs across the United States. Together, the two LEAGs inform the activities of the Centerand offer recipients of public health services the opportunity to shape them, ensuring they are responsive to their real-life needs. The LEAGs have also contributed to the Center’s dissemination work over the past three years. During the Center’s 2022 national conference, “The Public Health Opportunities and Challenges of Dementia Caregiving,” LEAG members led Mission Moments: engaging storytelling sessions that emphasized the importance of centering the voices of people with dementia and their caregivers in public health work. In addition, LEAG members have participated in Center webinars, sharing their personal experiences of living with mild cognitive impairment. Finally, both LEAGs have also helped identify priority topics for Center webinars, evaluated the Center’s website, and provided feedback and input on various PHCOE-DC resources to ensure they reflect the real-life experiences of dementia caregivers and are useful to health departments (Kline et al., 2025).

## Future methodological/conceptual considerations

The PHCOE-DC’s initial five-year funding cycle ended in September 2025, and 12 months of bridge funding were awarded to continue the Center’s work. Center leadership and its Executive Committee have identified several areas that could galvanize future iterations of the Center’s activities, alongside its robust dissemination efforts. Below is a summary of these ideas.

### Implementation strategies and outcomes

A major advance and guiding framework for the HBI and the BOLD Centers of Excellence is the HBI Road Map, 2023–2027 (Alzheimer’s Association & Centers for Disease Control and Prevention, 2023). The HBI Road Map provides a framework for public health action (see the paper by Roberts and colleagues in this issue). Over the past 2 decades, the Road Map has outlined numerous public health strategies to promote brain health, manage and address dementia and dementia risk, and support caregivers of individuals with dementia. Table 1 highlights selected Road Map action items that focus on supporting dementia caregivers. The creation of the HBI Road Map and its supporting frameworks is a considerable effort that requires input from public health professionals, scholars, persons living with dementia, and caregivers. An Implementation Guide is also provided to offer several activities to facilitate the adoption of selected Road Map Actions.

**Table 1.** Selected healthy brain initiative road map actions, dementia caregiving.

Outcome	Description
**Strengthen partnerships and policies**	
**P-3**	Partner across the community to promote *equitable access to services, supports and quality care* for people living with dementia and their caregivers.
**P-6**	Equip *policymakers* with information on risk reduction, the stigma associated with cognitive impairment and the impact of SDOH; and offer evidence-informed policy options across the life course.
**P-7**	Join ongoing coalitions and partnerships to prevent or remediate abuse, neglect and exploitation of people living with dementia.
**Build a diverse and skilled workforce**	
**W-1**	Provide evidence-informed training and informational resources for *primary health care providers* to facilitate culturally sensitive conversations about brain health with patients and caregivers across the life course.
**W-3**	Promote inclusion of the life course approach to brain health in *licensing, certification and continuing education* requirements for health care and allied professionals.
**W-4**	Strengthen training of *community health and direct service workers* about brain health across the life course to improve equitable care and quality of life for those living with cognitive decline and to support caregivers.
**W-5**	Partner with *public safety and emergency response agencies* to improve their ability to recognize and meet the needs of people living with cognitive decline and dementia.
**Measure, evaluate and utilize data**	
**M-1**	Support implementation of the BRFSS optional modules for Cognitive Decline and Caregiving, and use the data to develop and inform programs and policies.
**Engage and educate the public**	
**E-1**	*Engage diverse audiences* to develop *culturally responsive messaging* about brain health, cognitive decline, healthy aging and caregiving.
**E-2**	Disseminate culturally responsive messaging to *encourage conversations* about brain health, cognitive decline, healthy aging and caregiving.
**E-6**	Enhance communication with *people living with dementia, their families and caregivers* about how to access services, care and social supports.
**E-7**	Ensure *caregivers* have information, tools and resources about their vital role and ways to maintain their own *health and well-being*.

*Note*. This table was adapted from Healthy Brain Initiative: State and Local Road Map for Public Health, 2023–2027, by the Alzheimer’s Association and Centers for Disease Control and Prevention, 2023. P = develop policies & mobilize partnerships; W = assure a competent workforce; M = monitor & evaluate; E = educate & empower.

One research approach that could advance the uptake of Road Map Action items related to dementia caregiving is the application of implementation science methodologies. Implementation science is the study of strategies to adopt and integrate evidence-based interventions into clinical, community, healthcare, or other settings to improve individual and population outcomes (Brownson et al., 2023). Implementation science contributes three essential elements that can accelerate the successful adoption of HBI Road Map Actions: theories/models/frameworks (which provide explanations as to how/why implementation occurs successfully or not; Nilsen, 2020, p. 11), implementation strategies (i.e., approaches to effectively embed an evidence-based innovation into healthcare, organizational, community, or home settings; Leeman & Nilsen, 2020), and implementation outcomes (measures of the extent to which one or more strategies are successful when integrating innovation into real-world settings/routine care; Proctor et al., 2023). The incorporation of implementation science methodology will facilitate a greater understanding of success, as well as how and why specific strategies are more effective than others. Such insights will establish best practices across states and communities, and expedite the implementation of HBI Road Map actions.

### Further leveraging social determinants of health

The CDC defines SDOHs as “conditions where people are born, grow up/age, and live” (Centers for Disease Control and Prevention, 2024). The five domains of the CDC SDOH framework include: (1) economic stability; (2) educational access and quality; (3) healthcare access and quality; (4) neighborhood and built environments; and (5) social and community support. Others have incorporated systems and structures that influence people’s health regularly into SDOH frameworks, such as racism or other “isms.” The SDOH framework lends a public health perspective to designing interventions that improve health and reduce health disparities (Gaugler et al., 2023).

A significant opportunity is the continued alignment of dementia caregiving within an existing SDOH framework. As suggested in Figure 1, public health actions such as surveillance/identification, community partnerships to facilitate dementia-friendly communities, ensuring dementia-capable healthcare that incorporates care partner needs, and stigma reduction are all potential public health interventions that could target key SDOH-related mechanisms that, in turn, may improve dementia caregiver health. This multi-dimensional, holistic perspective may also help guide future descriptive research on dementia caregiving (in terms of how dementia caregiving influences and is influenced by SDOHs) as well as public health intervention evaluations and implementation efforts (Gaugler et al., 2023).

## Specific initiatives to advance the public health of dementia caregivers

### The Alter^TM^ Program

Faith-based organizations play a crucial role as important public health entities, particularly for traditionally underserved and underrepresented communities. A notable example of engagement with African American communities involving dementia and dementia caregiving is the Alter™ program, as described earlier. The PHCOE-DC has initiated important efforts to disseminate Alter™ to public health agencies and BOLD recipients through its toolkit development process (see above). As the PHCOE-DC looks toward the future, the further dissemination of Alter™ to public health agencies across the United States is viewed as a critical opportunity to support people living with dementia and their caregivers, as well as an approach to more effectively scale this program. Alter™ dissemination has primarily occurred at the FBO level, with heavy in-person engagement by the program developer, Dr Fayron Epps, and her team. This “high touch” approach will likely limit Alter™’s dissemination potential, particularly if, in future years, the program is culturally adapted and the number of potential adopters grows exponentially. Leveraging the preliminary work of the PHCOE-DC in engaging with the Mississippi Department of Health and the development of a public health agency-facing toolkit to facilitate engagement and partnership with FBOs (The BOLD Public Health Center of Excellence on Dementia Caregiving, 2025b), a template is now available for the PHCOE-DC to expand this work to multiple states, particularly BOLD Program recipients, to further disseminate Alter™ and more generally facilitate collaborations with FBOs to support dementia caregivers. Data on the toolkit’s utilization are forthcoming. As the Alter™ program continues to scale, key metrics will be systematically collected to evaluate its reach and impact on caregiver well-being, as well as its effectiveness and uptake in local support services (e.g., Area Agencies on Aging, Meals on Wheels).

### Crowdsourcing dementia care services and supports

Community asset mapping is a method that public health agencies have employed to address various community-based health needs (Luo et al., 2023). Community asset mapping identifies resources and services in the community, ranging from formal, well-identified organizations to informal, “intangible” resources that are less well-known or advertised. A core aspect of community asset mapping is that communities identify resources to leverage the accumulated knowledge and experience of community members about where they live, living with a health condition, and/or caring for someone with such a condition (Luo et al., 2023; Ruggiano et al., 2024). Specifically, “crowdsourcing” community dementia care services and supports (where volunteers enter relevant information about services and supports) to create a dynamic resource may provide an online tool/mobile application that could harness the mutual support often available among dementia caregivers living in a local community (Ruggiano et al., 2024).

Alabama Caregiver Connect is developing community asset mapping software to support dementia caregiver resources and services throughout the state. Volunteered geographic information, collected through a crowdsourcing protocol, will populate a dementia resource and service map for caregivers and healthcare professionals. Alabama Caregiver Connect is currently undergoing feasibility testing with local Alabamians to populate the resource map when seeking information (­Ruggiano et al., 2024). There are multiple issues to consider as this technology is refined and developed, including accuracy (e.g., the need for algorithms to ensure accuracy and reduce the “noise” of crowdsourced data to reduce misleading/false information shared; ensure that the tool remains dynamic and updated so that changes in service availability, accessibility, and quality are readily available for dementia caregivers) (Ruggiano et al., 2024). However, a dynamic “map” of dementia caregiver prevalence that is detailed enough to be useful for local communities and offers information about available services and supports for dementia caregivers could likely be deployed across states.

### Building community, public health education, and engagement

The PHCOE-DC, as part of supplemental CDC support, offered a highly successful national conference on the public health challenges and opportunities for dementia caregiving in 2022. Given the success of this event and the collaboration it fostered, offering a similar national conference annually will help to further build mutual learning and partnerships across public health agencies as they implement actions to support dementia caregivers in their jurisdictions.

### Dementia-friendly communities and memory cafés

One initiative that public health agencies can align their efforts with for dementia and dementia caregiver support is Dementia Friendly Communities, administered under the auspices of Dementia Friendly America^®^. A community-led movement, Dementia Friendly America^®^ has as its mission to make living with dementia or caring for someone with dementia more meaningful and less stigmatizing through the mobilization of community connections and supportive built environments. At least 350 communities are working to make their neighborhoods and environments more dementia-friendly, according to Dementia Friendly America^®^ (USAging, 2024).

An approach that could advance the dementia friendliness of a community is a Memory Café (MC). The primary goal of MCs is to provide socialization and meaningful activities for individuals living with dementia, their care partners, friends, and professional caregivers. Memory Cafés originated in the Netherlands in 1997 and have since been disseminated worldwide as a low-cost approach to strengthen social connections for individuals living with dementia and their caregivers in the community. It is estimated that around 900 MCs in the United States serve ∼45,000 people living with dementia (Basting et al., 2024). However, given their informal nature and structure, it is likely that many more exist. Until recently, no effort or organization has been in place to coordinate MCs across the United States and ensure quality training and program delivery. The Memory Café Alliance is a new national initiative that was founded and is led by a grassroots movement of MCs to promote and sustain the MC model, provide opportunities for collaboration among MC providers to share programming ideas, and improve training to ensure quality. Dementia Friendly America^®^ is a lead partner of the Memory Café Alliance.

Engagement with the Memory Café Alliance aligns with the HBI Road Map’s Actions and the role of public health agencies in disseminating information and supporting people living with chronic conditions and their care partners. Similar to Alter^TM^, the PHCOE-DC could facilitate linkages between public health agencies and local MCs by: a) connecting them with the Memory Café Alliance; b) developing toolkits to identify specific actions and strategies for public health agencies to support and disseminate MCs, and 3) highlighting successful approaches that support the broad dissemination of the MC model by public health across the United States.

## Conclusion

Alzheimer’s disease and related dementias are incredibly challenging for people living with these conditions. However, Alzheimer’s disease is also a disease of context, meaning that the insidious, progressive nature of dementia significantly influences the emotional, psychological, social, physical, and financial health of the individuals and organizations who care for people living with ADRD. The significant increase in the number of people living with ADRD, the sheer number of unpaid relatives, friends, and others who provide the bulk of care to people living with dementia, and the disparities in care access and outcomes among caregivers in minoritized, under-resourced communities make dementia caregiving a pressing public health concern. The BOLD PHCOE-DC, in collaboration with its HBI partners, has provided direct support and disseminated resources, tools, and information to public health agencies nationwide, helping them address the needs of dementia caregivers. If ongoing federal funding for the BOLD PHCOE-DC and its HBI partners is made available (for more details, please see McGuire, 2025 in this issue), in its next iteration the PHCOE-DC will continue its robust national dissemination activities alongside the championing and implementation of specific public health actions (e.g., public health dissemination support of Alter^TM^, crowdsourcing approaches, national public health conference, Memory Cafés) to support the vast, unpaid network of dementia caregivers in the United States.

## Data Availability

The authors do not report data, and therefore, the pre-registration and data availability requirements are not applicable.
